# Case Report: IL-21 and Bcl-6 Regulate the Proliferation and Secretion of Tfh and Tfr Cells in the Intestinal Germinal Center of Patients With Inflammatory Bowel Disease

**DOI:** 10.3389/fphar.2020.587445

**Published:** 2021-01-26

**Authors:** Youguang Yang, Xiaodan Lv, Lingling Zhan, Lan Chen, Hui Jin, Xinping Tang, Qingqing Shi, Qiyuan Zou, Jiqiao Xiang, WeiWei Zhang, Zhaojing Zeng, Haixing Jiang, Xiaoping Lv

**Affiliations:** ^1^ Department of Gastroenterology, The First Affiliated Hospital of Guangxi Medical University, Nanning, China; ^2^ Department of Clinical Experimental Medicine, The First Affiliated Hospital of Guangxi Medical University, Nanning, China

**Keywords:** Bcl-6, follicular helper T cells, follicular regulatory T cells, germinal center, IL-21, inflammatory bowel disease

## Abstract

**Objective**: This study aimed to investigate the effect of interleukin (IL)-21 and B cell lymphoma protein-6 on germinal center follicular helper T (Tfh) cells and follicular regulatory T (Tfr) cells and its relationship with the clinical features of inflammatory bowel disease (IBD).

**Methods**: The expression of peripheral blood cytokines IL-21 and Bcl-6 mRNA was detected by reverse transcription–polymerase chain reaction. The distribution characteristics of Tfh and Tfr cells were detected using the triple immunofluorescence staining analysis.

**Results**: The expression of IL-21 and Bcl-6 mRNA was upregulated in the ulcerative colitis (UC) and Crohn disease (CD) groups compared with that in the control group. Triple immunofluorescence staining showed that the number of Tfh cells in the intestinal germinal center obviously increased in the UC and CD groups compared with that in the control group, whereas the number of Tfr cells reduced.

**Conclusion**: This study suggested that the Tfr and Tfh cells might be involved in the regulation of IBD. Bcl-6 and IL-21 can regulate the Tfh/Tfr ratio in the intestinal germinal center, promoting the occurrence and development of IBD.

## Introduction

Inflammatory bowel disease (IBD) is characterized by inflammation and abnormal bowel patterns. However, its etiology and pathogenesis are still not clear, possibly due to intestinal microbial infection or physicochemical factors (such as certain foods and drugs), coupled with a variety of factors related to individual genetic susceptibility, or caused by excessive activation of intestinal mucosal immune response (Baumgart and Sandborn, 2007) IBD includes ulcerative colitis (UC) and Crohn disease (CD). The main difference between UC and CD is that UC is mainly located in the colon and rectum, while CD can affect the entire digestive tract (Baumgart et al., 2012; Ordás et al., 2012). Two new types of T helper cells have been found in the germinal center of lymphoid tissues: follicular helper T (Tfh) cells and follicular regulatory T (Tfr) cells, which differentiate and proliferate in lymphoid follicles. (Deenick and Ma, 2011; Linterman et al., 2011) These two types of cells are closely related to various autoimmune diseases (Wang et al., 2014; Dhaeze et al., 2015). Despite minimal research on the Tfr/Tfh ratio in patients with IBD, some scholars (Ozaki et al., 2002; Eto et al., 2011) reported that changes in the levels of IL-2 and IL-6 in the local microenvironment of IBD might be involved in the differentiation of Tfh cells and the abnormal expression of transcription factors such as Bcl-6 and c-MAF. Treg cells may also be involved in the abnormal regulation of Tfh cells. Based on the characteristics of IBD with excessive immune damage and autoimmune response, it is reasonable to speculate that Tfh and Tfr cells are involved in the progression of IBD. The proportion of Tfh and Tfr cells in the peripheral blood of patients with IBD and healthy population was detected by flow cytometry to determine the correlation between Tfh/Tfr ratio and IBD. The expression of IL-21 and Bcl-6 was assessed by reverse transcription–polymerase chain reaction (RT-PCR) in patients. Moreover, triple immunofluorescence staining analysis was performed to detect the distribution characteristics of Tfh cells in the germinal center and Tfr cells in the intestinal tissue of patients with IBD by illustration there specia cell phenotype CD57/FoxP3. The present study might provide a theoretical basis for the clinical treatment of IBD by the intervention of Tfh and Tfr cells.

## Materials and Methods

### Materials

Blood samples were collected from 134 patients with IBD in the Department of Gastroenterology, the First Affiliated Hospital of Guangxi Medical University, China. Among them, 79 were males and the remaining 55 were females. The age ranged from 21 to 65 years, with an average age of 38.12 ± 7.23 years colorectal inflammation tissue of 111 patients were collected by endoscopy. Among them, 69 were males and the remaining were females. The age ranged from 21 to 68 years, with an average age of 37.08 ± 6.03 years. In addition, 90 cases of blood samples and 80 cases of colorectal tissue samples were collected from healthy controls. The subjects were healthy people who had been treated in the First Affiliated Hospital of Guangxi Medical University at the same time, who had excluded tumor, autoimmune disease and family history of IBD. There was no significant difference in age and gender between IBD group and healthy control group (*p* > 0.05). All specimens obtained were approved by ethics committee of the First Affiliated Hospital of Guangxi Medical University, and all patients were informed and agreed.

### Methods

#### Expression of Peripheral Blood Cytokines IL-21 and Bcl-6 mRNA Was Detected by RT-PCR in Patients With IBD and Healthy Controls

The total RNA was extracted from the peripheral blood using chloroform and TRIzol solution (TaKaRa Bio Inc., Shiga, Japan). A first-strand cDNA synthesis was performed with 1 μg of total RNA. The cDNA samples were thereafter amplified in the ABI Prism 7500 Sequence Detection System (Applied Biosystems, MA, United States) for 40 cycles (95°C for 3 s and 60°C for 34 s) with specific oligonucleotide primers (TaKaRa Bio Inc.). Each sample was analyzed in triplicate, with β-actin used for normalization. The relative quantification of target genes was determined using the ΔΔCT method. The primers used in RT-PCR analyses are listed in Table 1.

**TABLE 1:** Primer sequences for PCR.

Gene name	Direction	Sequences (5ʹ–3ʹ)
IL-21	Forward	CAC​AGA​CTA​ACA​TGC​CCT​TCA​T
	Reverse	GAA​TCT​TCA​CTT​CCG​TGT​GTT​CT
Bcl-6	Forward	CGGAAGGGTCTGGTTAG
	Reverse	TGA​GCA​CGA​TGA​ACT​TGT​AT
Β-actin	Forward	CCC​ATA​CCC​ACC​ATC​ACA​CC
	Reverse	GAG​AGG​GAA​ATC​GTG​CGT​GAC

β-actin; IL, interleukin; PCR, polymerase chain reaction.

#### Triple Immunofluorescence Staining Analysis of the Distribution Characteristics of Tfh/Tfr Ratio in the Germinal Center

IBD intestinal tissue was taken and fixed with paraformaldehyde. The paraffin-embedded sections were stained with immunohistochemistry. The tissue sections were washed with PBS, water, and then skimmed milk. Two groups of three fluorescent-labeled antibodies were added at room temperature: the first group comprised the primary antibodies to CD4 (BioLegend, United States) , CXCR5, and FOXP3 (eBioscience, United States); the second group comprised the primary antibodies in three different colors: green fluorescent protein labeling, Cy3-labeled red fluorescence, and AMCA blue fluorescence. The secondary antibodies were stained in three different colors, representing different Tfr and Tfh cells. The results were analyzed using average optical density values.

## Statistical Analysis

All data were expressed as mean ± standard deviation. Comparisons between groups were performed using one-way analysis of variance followed by the Student–Newman–Keuls post hoc test. *p* values less than 0.05 were considered statistically significant. All the statistical analyses were performed using the statistical software package SPSS version 16.0 (SPSS Inc., IL, United States).

## Results

### Effect of IBD on the mRNA Expression of IL-21 and Bcl-6

**FIGURE 1:**
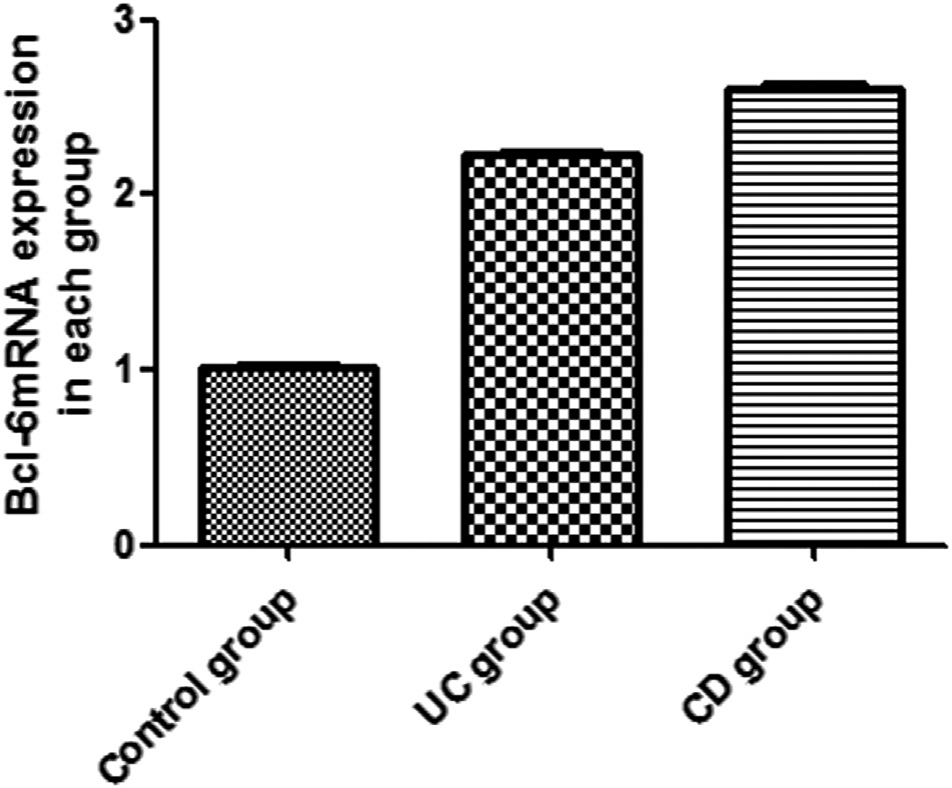
Expression of Bcl-6 mRNA in the peripheral blood of UC and CD groups.

**FIGURE 2:**
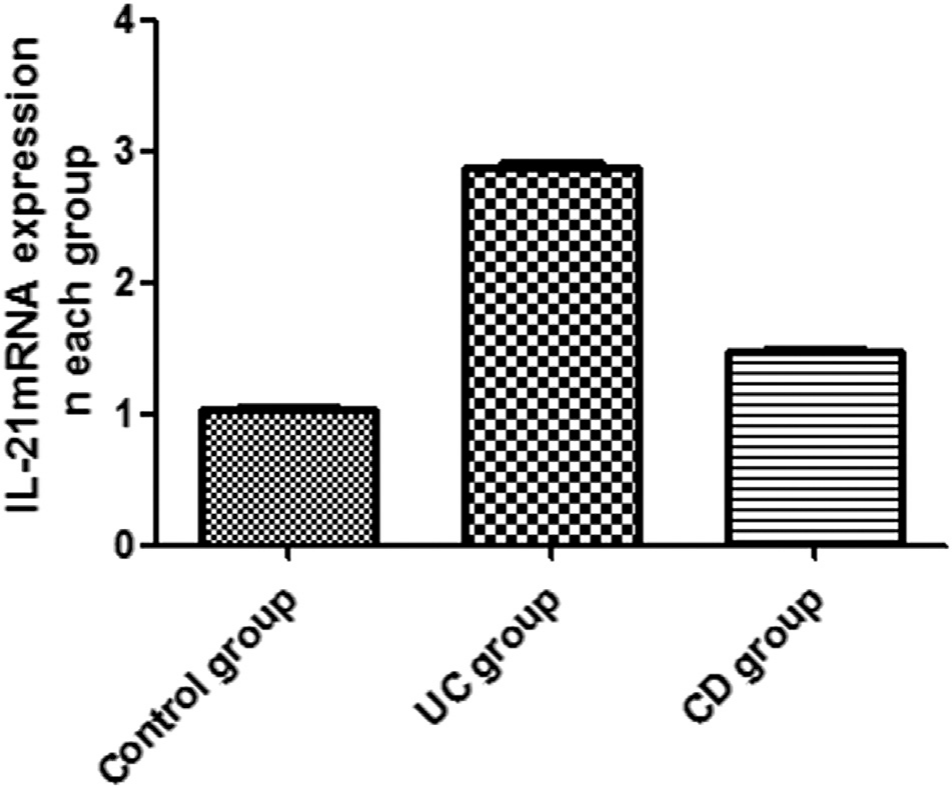
Expression of IL-21 mRNA in the peripheral blood of UC and CD groups.

The expression of IL-21 and Bcl-6 mRNA was examined in the peripheral blood by RT-PCR (Figures 1, 2). The expression of IL-21 and Bcl-6 mRNA was low in the control group, while it significantly increased in the UC and CD groups (*p* < 0.05). The reason probably is that Tfh can secrete IL-21R in an autocrine manner at the same time, IL-21R can also stimulate the production of Bcl-6, which can also secrete Tfh. Therefore, it was hypothesized that IL-21 and Bcl-6 played an important role in the pathogenesis of IBD.

### Triple Immunofluorescence Staining Analysis of the Distribution Characteristics of Tfh/Tfr Ratio in the Germinal Center

**FIGURE 3:**
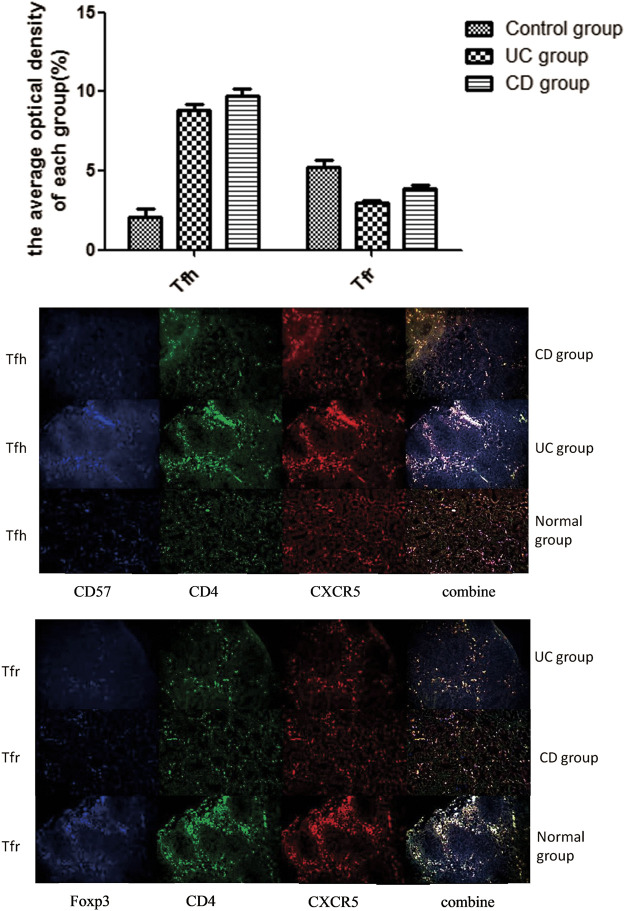
Tfh and Tfr triple immunofluorescence staining (×20).

Triple immunofluorescence staining was performed to analyze the distribution characteristics of Tfh/Tfr ratio in the germinal center of intestinal tissues (Figure 3). Red fluorescence indicated CXCR5; green fluorescence indicated CD4; and blue fluorescence indicated FOXP3/CD57. The coincidence of white fluorescence with red, blue, or green fluorescence indicated that the site was Tfh or Tfr cells. When the three channels were coincident, the white light appeared in the germinal center of the lymph nodes. We used the average of optical density to evaluate the intensity of cell staining in each groupStaining of the intestinal tissue samples from the normal control group showed that the staining intensity of Tfr cells in the germinal center was obviously upregulated compared with the UC and CD groups (*p* < 0.05). However, the staining intensity of Tfh cells in the germinal center from the UC and CD groups significantly increased compared with the control group (*p* < 0.05).

## Discussion

IBD is a disease with autoimmune characteristics because of immune response imbalance. The incidence of IBD is continuously rising; however, its etiology and pathogenesis are not clear.

The Tfh cells are regulated by B cells, Inducible costimulator, SLAM-associated protein, and PD-1, whose surface marker is CD4+CD57+CXCR5. They can secrete IL-21, IL-6, and IL-27 (Auderset et al., 2013). IL-21, a T-cell–derived cytokine, is produced in excess in inflammatory bowel diseases, which can be highly expressed in intestinal mucosa of IBD patients (Chelsky et al., 2007). The main function of Tfh cells is to form B cells with high-affinity antibodies and lack of differentiation into autoreactive plasma cells and memory B cells with high affinity and long-term protective effect against infection. Tfh cells also play a key role in promoting the formation of germinal center, immunoglobulin class switching, and maintaining long-term humoral immune response (Cannons et al., 2013). It has been found that IL-21 and Bcl-6 are the key factors regulating the development and function of Tfh cells. The research shows that Tfh cells form but decline faster in the absence of IL-21 (Linterman et al., 2010). The most probably reason is that IL-21 is involved in the differentiation of B cells, which are produced by the Tfh cells and play a key role in the survival of Tfh cells. It is involved in the transformation of CD4+T cells into Tfh cells, which is essential for the growth and development of Tfh cells (Yu et al., 2015).

Tfr is a newly discovered regulatory T cell subgroup. Tfr surface markers, including Tfh cell-related molecules, play an essential role in the regulation of germinal center response (Linterman et al., 2011; Linterman et al., 2012). Tfr cells can express CD8, CXCR5, Bcl-6, and FOXP3, and are located in the germinal center, inhibiting the immune response (Deaglio et al., 2007). Tfr cells are characterized by the overlap of surface phenotype and Tfh cells, so that Tfr and Tfh have dual functional characteristics. Tfr cells can regulate the number of Tfh cells and the response of germinal centers (Szanya et al., 2002; Borsellino et al., 2007; Deaglio et al., 2007). If FOXP3+Bcl-6+Treg lacks in the follicles, the germinal centers lose the role of inhibiting the immune response, resulting in the secretion of antibodies from a large number of B cells. Therefore, it is believed that FOXP3+Bcl-6+ Treg is an autoimmune regulatory signal (Wollenberg et al., 2011). However, Tfr and Tfh cells have the same phenotypic characteristics, which may be related to the germinal centers (Chung et al., 2011). A certain number of Tfr cells exist in the circulation, which can be activated by CD28 and ICOS and inhibited by PD-1 and PD-L1 (Sage et al., 2012). The specific inhibition needs further investigation. A recent study showed that Tfr cells mediated Tfh cell and B cell responses, regulating the germinal center reaction. Therefore, Tfr cell dysregulation is critical to the development of autoimmune diseases (Sage and Sharpe, 2016).

Tfh can secrete IL-21R in an autocrine manner, which promotes the proliferation of Tfh cells with high expression of CXCR5 and ICOS and their migration to lymph nodes and germinal centers (Sarra et al., 2012; Spolski et al., 2012). However, Tfh cells not only increase the expression of Bcl-6 but also increase the expression of Tfh surface protein gene. The reason may be that Bcl-6 is necessary for the differentiation of CD4+T cells into Tfh cells *in vivo* (Johnston et al., 2009). The high expression of Bcl-6 can induce the formation of Tfh phenotype cells (such as CXCR5, PD-1, and CXCR4) (Nurieva et al., 2009). The initial T cells receive dendritic cells presenting antigen and ICOS stimulation signals and begin to express Bcl-6 at high levels (Choi et al., 2013). It has been confirmed that transcription factors and regulatory factors Bcl-6 and IL-21 are important factors in the regulation of Tfh differentiation (Wollenberg et al., 2011; Ding et al., 2014). The changes in the expression of IL-21 and Bcl-6 mRNA in the peripheral blood were measured in the present study to evaluate the numbers of Tfh cells. The results showed an increase in the expression of IL-21 and Bcl-6 mRNA, suggesting an increase in the number of Tfh cells. The lack of Tfr cells might be due to the decrease in STAT3 in Treg cells (Hao et al., 2016). The loss of Tfr cells and the corresponding decrease in the cell phenotype FOPX3 lead to a decrease in the number of Treg cells (Aloulou et al., 2016). An increase in the number of Tfh cells promotes the activation of Bcl-6/IL-21, leading to the occurrence and development of IBD.

This study showed two kinds of cells in patients with IBD having abnormal regulation and differentiation: Tfh and Tfr. It was suggested that Tfh and Tfr might be involved in the immune regulation of IBD. These results also suggested that Bcl-6/IL-21 could regulate the changes in Tfh/Tfr ratio and promote the occurrence and development of IBD.

In summary, this study suggested that Tfh cells helped B cells in germinal center formation and produced high-affinity antibodies. However, Tfr cells inhibited B cells, which secreted IL-21, thereby reducing the inflammatory reaction. Tfr cells play a key role in negative immune regulation in the pathogenesis of IBD. This study may provide a theoretical basis for future studies on the pathogenesis and clinical treatment of IBD.

## Data Availability Statement

The raw data supporting the conclusions of this article will be made available by the authors, without undue reservation.

## Ethics Statement

The studies involving human participants were reviewed and approved by Medical Ethics Committee of the First Affiliated Hospital of Guangxi Medical University. The patients/participants provided their written informed consent to participate in this study.

## Author Contributions

XL and LZ: designed and supervised the research. YY, XL, LC, QS, QZ, and JX: performed the experiments. YY, HJ, XT, and WZ: acquired and analyzed the data. YY: drafted and wrote the manuscript. HJ and XL: revised the manuscript for important intellectual content. All the authors have read and approved the final version to be published.

## Funding

National Natural Science Foundation of China (No. 81860104, 81460114 and 81860120). Natural Science Foundation of Guangxi Zhuang Autonomous Region (No. 2017GXNSFAA198299 and 2017GXNSFBA198134). The Development and Application of Medical and Health Appropriate Technology Project in Guangxi Zhuang Autonomous Region (No. S2018049). The Youth Science Foundation of Guangxi Medical University (No. GXMUYSF201913 and GXMUYSF201908). The Self-financing Project of Health Commission of Guangxi Zhuang Autonomous Region (No. Z20200398).

## Conflict of Interest

The authors declare that the research was conducted in the absence of any commercial or financial relationships that could be construed as a potential conflict of interest.
